# Ginger Extract Decreases Susceptibility to Dextran Sulfate Sodium-Induced Colitis in Mice Following Early Antibiotic Exposure

**DOI:** 10.3389/fmed.2021.755969

**Published:** 2022-01-05

**Authors:** Xinghong Zhou, Xiaoyu Liu, Qiuxing He, Ming Wang, Hanqi Lu, Yanting You, Liqian Chen, Jingru Cheng, Fei Li, Xiuqiong Fu, Hiu Yee Kwan, Lin Zhou, Xiaoshan Zhao

**Affiliations:** ^1^Department of Traditional Chinese Medicine, Zhujiang Hospital of Southern Medical University, Guangzhou, China; ^2^Syndrome Laboratory of Integrated Chinese and Western Medicine, School of Chinese Medicine, Southern Medical University, Guangzhou, China; ^3^Department of Nephrology, The First Affiliated Hospital of Zhengzhou University, Zhengzhou, China; ^4^Department of Traditional Chinese Medicine, The Affiliated Ganzhou Hospital of Nanchang University, Ganzhou, China; ^5^School of Chinese Medicine, Hong Kong Baptist University, Hong Kong, China; ^6^Department of Endocrinology, Nanfang Hospital, Southern Medical University, Guangzhou, China

**Keywords:** ginger, antibiotic, childhood, colitis, microbiota

## Abstract

**Background:** Intestinal microbial colonization in early life plays a crucial role in immune development and mucosal homeostasis in later years. Antibiotic exposure in early life increases the risk of inflammatory bowel disease (IBD). Ginger acts like a prebiotic and has been used in traditional Chinese medicine for colitis. We investigated the protective effect of ginger against dextran sulfate sodium (DSS)-induced colitis in mice exposed to antibiotic in their early years.

**Methods:** A weaned mouse model exposed to azithromycin (AZT) for 2 weeks was used to mimic antibiotic exposure in childhood among humans. A diet containing ginger extract was administered to mice for 4 weeks after antibiotic exposure. The susceptibility to DSS-induced colitis was evaluated in terms of weight loss, disease activity index (DAI) score, colon length, colitis biomarkers, and intestinal barrier function. The gut microbiota was analyzed in terms of 16S rRNA levels.

**Results:** Ginger extract prevented weight loss, colon shortening, inflammation, and intestinal barrier dysfunction in mice exposed to antibiotics in early life. Ginger increased the bacterial diversity and changed the abundance of bacterial belonging to family *Peptococcaceae* and *Helicobacter* species to modulate microbiota structure and composition adversely affected by early antibiotic exposure.

**Conclusion:** Ginger has a protective effect in potentially decreasing the susceptibility to colitis in mice exposed to antibiotics early in life.

## Graphical Abstract

Ginger extract decreases susceptibility to dextran sulfate sodium-induced colitis in mice following early antibiotic exposure. 
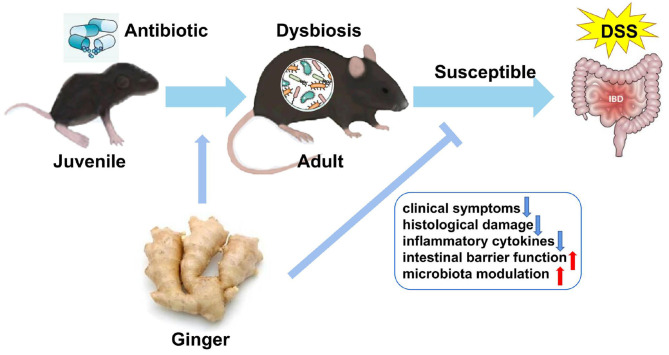

## Introduction

Inflammatory bowel disease (IBD), mainly including ulcerative colitis (UC) and Crohn's disease (CD), is characterized by chronic intestinal inflammation. The incidence and prevalence of IBD have increased worldwide in the recent decades, indicating its emergence as a global public health challenge (1). The lack of adequate and appropriate treatment prompted the search for alternative therapeutic strategies. Although the etiology of IBD is not fully understood, current evidence indicates that a complex interaction among genetic susceptibility, environmental factors, intestinal microbiota, and immune response is involved in the pathogenesis of IBD (2). The role of intestinal microbiota in IBD pathogenesis has recently been highlighted (3).

Antibiotic exposure is one of the most common factors leading to intestinal microbiota dysbiosis. Antibiotic use accounts for a large proportion of children's prescriptions. In the United States, about 44.5 million courses were prescribed to children under the age of 10 years, with the highest prescription rate found in the first 2 years of life. Penicillins and macrolides were the most common antibiotic categories prescribed in children, whereas azithromycin (AZT), the typical drug belonging to the family of macrolides, was the most frequently prescribed antibiotic agent (4, 5).

Early childhood is considered a dynamic phase of the intestinal microbial ecosystem, which is easily shaped by environmental factors. Disruption of microbiota by antibiotic exposure in this sensitive period may be associated with long-term adverse effects (6, 7). Epidemiological and experimental studies have reported an association between early-life exposure to antibiotics and an increased risk of asthma, obesity, and impaired antibody response to vaccination (8–10). A strong association between antibiotic use and CD in childhood was found in a prospective study (11). Peripartum antibiotics promoted gut dysbiosis, immune dysfunction, and IBD in offspring in animal studies (12). Restoration of gut microbial levels reduced the risk of colitis associated with antibiotic-induced gut dysbiosis in mice (13).

Since early antibiotic exposure may increase the susceptibility to diseases later, it is meaningful to identify effective measures to prevent these changes. Ginger (*Zingiber officinale)* rhizomes are not only used as a food but are also a common traditional Chinese medicine administered to treat various diseases. Recent studies demonstrated that ginger and ginger extracts exhibit antiinflammatory effects and regulate intestinal microbiota (14, 15). Novel ginger-derived nanoparticles have been shown to reduce and prevent acute colitis and colitis-associated cancer and enhance intestinal repair (16, 17). Ginger exosome-like nanoparticles (ELNs) are preferentially taken up by specific bacteria, resulting in changes in bacterial composition and localization, and also host physiology, notably enhancing gut barrier function to alleviate colitis (18). These findings suggest the role of ginger in the modulation of microbiota and treatment of IBD. However, the anticolitis activity of ginger against intestinal microbiota in early antibiotic exposure mice has yet to be clearly established.

We hypothesized that early-life antibiotic exposure may have long-term effects in predisposing the host to IBD in later years by altering intestinal microbiota. Administration of ginger after early antibiotic exposure may decrease the susceptibility to IBD. To test this hypothesis, we analyzed the impact of early-life antibiotic exposure on the risk of IBD in later years and tested the regulatory effect of ginger using a juvenile mouse model mimicking childhood antibiotic exposure in humans.

## Materials and Methods

### Chemicals and Materials

Dextran sulfate sodium (DSS, MW:36,000–50,000 Da) was purchased from MP Biomedicals (CA, USA). AZT was purchased from Aladdin Bio-Chem Technology Co., Ltd (Shanghai, China). An AZT dose of 50 mg/kg was used in this study to ensure therapeutic plasma levels in the mice; it is the highest dose administered to mice in a multidose regimen (19). Ginger extract (number 1708001W), which meets the quality criteria of granules used in Chinese herbal medicine prescriptions (T-SZ-PC-0390-003), was purchased from China Resources Sanjiu Medical and Pharmaceutical Company (Shenzhen, China). All feeds used in this study were purchased from Guangdong Medical Laboratory Animal Center (Foshan, China). The feeds were sterilized with Co60 (25 kGy) radiation. A standard AIN-93G diet was used as a normal basic diet. Ginger diet was modified from AIN-93G by replacing 10-g corn starch in the original formula with equal amounts of ginger extract per 1 kg (Table 1). Fecal occult blood test kit was ordered from Nanjing Jiancheng Bioengineering Institute (Nanjing, China). ELISA kits for assaying the contents of tumor necrosis factor-α (TNF-α), interleukin-1β (IL-1β), interleukin-6 (IL-6), and interleukin-10 (IL-10) in the colon were purchased from Dakewe Biotech Co., Ltd. (Shenzhen, China). All other reagents used were of analytical grade. Antibodies against zonula occludens 1 (ZO-1) (rabbit, AF5145), claudin-1 (rabbit, AF0127), and β-actin (mouse, BF0198) were purchased from Affinity Biosciences (OH, USA). CD-68 antibody (rat, ab53444) was purchased from Abcam (Cambridge, England).

**Table 1:** Composition of experimental diets (g).

**Ingredient**	**Normal diet (AIN-93G)**	**Ginger diet**
Corn starch	397.5	387.5
Casein	200	200
Dextrinized corn starch	132	132
Sucrose	100	100
Soybean oil	70	70
Fiber	50	50
AIN-93G-MX	35	35
AIN-93G-VM	10	10
Ginger	0	10
L-Cystine	3	3
Choline chloride	2.5	2.5

### UPLC-QTOF-MS Analysis of Ginger Extract

The ginger extract was analyzed with ultra-performance liquid chromatography-quadrupole time-of-flight mass spectrometry (UPLC-QTOF-MS). The extract was separated on a HSS T3, 1.8 μm (2.1 mm × 100 mm) column and eluted with mobile phases of acetonitrile (A) and 0.25% formic acid in water (B) in gradient mode. The proportion of acetonitrile varied from 20 to 90% in 24 min (0–5 min, 20–30% A; 5–8 min, 30–40% A; 8–10 min, 40–45% A; 10–24 min, 45–90%) at a flow rate of 0.3 ml·min^−1^, with each injection volume was set to 5 μl. The MS data were acquired on an AB SCIEX Triple TOF 5,600 (AB sciex Pte. Ltd., Singapore). The MS parameters were as follows: interface, negative electrospray ionization (ESI); gases one and two, nitrogen 55 psi; curtain gas, nitrogen 40 psi; source temperature, 400 C; ion spray voltage, 5,500 V; declustering potential, 100 V and collision energy, 45 eV. The Peakview (version 2.0, AB SCIEX) was employed for the analyses. A representative chromatogram of ginger is presented in Supplementary Figure S1. A total of 16 components were identified according to their retention times (Supplementary Table S1).

### Animals

Three-week-old C57BL/6 male mice were purchased from Guangdong Medical Laboratory Animal Center (Guangzhou, China). Mice were maintained under an automated 12-h light–dark cycle at a controlled temperature of 22°C ± 2°C, and a relative humidity of 50–60% with *ad libitum* access to a standard dry diet and tap water. The study was approved by the Standards for Animal Ethics in the Guangzhou Institute of Sport Science (GZTKSGNX-2016-4) and performed in accordance with the relevant experimental animal guidelines and regulations.

### Experimental Design

**Figure 1:**
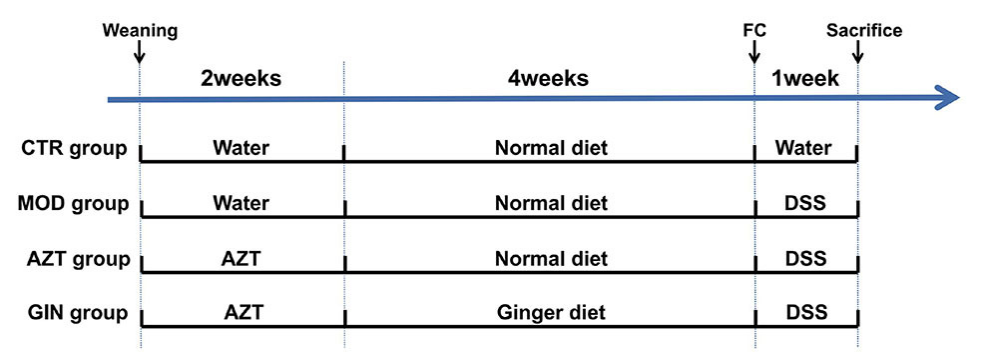
Schematic diagram summarizing the timeline of experimental procedures. Three-week-old C57BL/6 male mice were randomly assigned to four groups (*n* = 8–10): control (CTR) group; model (MOD) group; AZT group; and ginger (GIN) group. FC, fecal collection.

Juvenile C57BL/6J male mice were randomly assigned to four treatment groups (8–10 mice/group) immediately after weaning (Figure 1). The control (CTR) group was vehicle-treated and the model (MOD) group was given drinking water containing 2.5% DSS only to induce colitis. The AZT group received 2.5% DSS after the daily administration of AZT (50 mg/kg/day, dissolved in drinking water) for 2 weeks. The ginger (GIN) group received a ginger diet *ad libitum* for 4 weeks after 14 days of AZT exposure and then treated with 2.5% DSS for 7 days to induce colitis. Fecal samples were collected from model, AZT, and GIN groups before the administration of DSS. The samples were stored at −80°C for further analyses. Body weight, stool consistency, and stool bleeding were recorded daily during DSS treatment period.

### Evaluation of Disease Activity Index

Body weight, stool consistency, and stool bleeding were recorded daily. Disease activity index (DAI) was determined by dividing the combined scores of body weight loss, stool consistency, and stool bleeding, by three (Supplementary Table S2). Each score was determined as follows: change in body weight loss (0: none, 1: 1–5%, 2: 5–10%, 3: 10–15%, 4: > 15%), stool consistency (0: normal, 1–2: loose, 3–4: diarrhea), and stool bleeding (0: negative, 1: +, 2: ++, 3: +++, 4: ++++). Weight loss was calculated as the percent difference between the original body weight (day 0) and the body weight on any particular day. Blood in stool was detected using the fecal occult blood test kit according to the manufacturer's protocols. In addition, the colon length between the caecum and proximal rectum was measured.

### Histologic Examination

Paraffin-embedded tissue sections of the colon were stained with H&E for light microscopic examination to assess colon injury and inflammation. A modified combined scoring system (Supplementary Table S3) including degree of inflammation (scale of 0–3) and crypt damage (0–4), percentage of inflammation (0–4), and depth of inflammation (0–3) was used to assess colitis induced by DSS. The total score ranged from 0 (normal) to 14 (severe colitis).

### Electron Microscopy

Murine colon tissues were fixed in 3% glutaraldehyde, postfixed in 2% osmium tetroxide, and embedded in epoxy resin. Sections were stained with lead citrate and uranyl acetate and were viewed and photographed with an electron microscope (Hitachi, Japan).

### Immunohistochemical Staining

Paraffin-embedded colon sections were deparaffinized. After unmasking antigens, colon sections were blocked with 5% bovine serum albumin (BSA) and immunostained with anti-ZO-1 antibody or CD68 overnight at 4°C. Following immunostaining, sections were washed three times with PBS and then incubated with Alexa Fluor 488 (1:200, Beyotime, China) or Alexa Fluor 568 (1:200, Abcam, England) conjugated secondary antibody for 2 h at room temperature in the dark. Sections were then mounted with a medium containing 4,6-diamidino-2-phenylindole (DAPI) for nuclear counterstaining and observed by fluorescence microscopy.

### Western Blot Analysis

Colons were excised and washed thoroughly with PBS, homogenized in RIPA buffer containing protease inhibitors, incubated for 20 min at 4°C, and centrifuged for 20 min, 14,000 rpm at 4°C. Protein extracts were isolated from colon tissue. Samples were separated on 10% acrylamide gels and transferred onto polyvinylidenedifluoride (PVDF) membranes. The membranes were blocked with 5% (w/v) BSA in Tris-buffered saline/0.05% Tween-20 (TBST) at room temperature for 2 h in a covered container and incubated overnight at 4°C with primary antibodies in blocking buffer. The next day, membranes were washed with TBST (3^*^10 min) and incubated with a secondary goat antimouse or goat antirabbit IgG horseradish peroxidase (HRP) antibody (1: 10,000 dilution) diluted in 5% (w/v) dry nonfat milk in TBST for 1 h at room temperature. Finally, membranes were washed with TBST (3^*^10 min) and detected *via* electrochemiluminescence (ECL). .

### Enzyme-Linked Immunosorbent Assays

The levels of inflammatory cytokines (TNF-α, IL-1β, IL-6, and IL-10) in the mouse colon were determined with ELISA kits. The intestinal tissues were homogenized on ice in NP40 lysis buffer (Beyotime Biotechnology, China). The homogenates were quantified using the BCA assay (Beyotime Biotechnology, China). Tissue homogenates were collected for the determination of TNF-α, IL-1β, and IL-6 concentrations according to the manufacturers' instructions.

### Fecal DNA Extraction and 16S rRNA Gene Amplicon Analysis

Fecal samples from all mice were collected before the administration of DSS and stored at −80°C. Bacterial DNA was extracted from mouse fecal samples using a QIAamp Fast DNA Stool Mini kit (Qiagen, California, USA) according to the manufacturer's protocols. Purity was determined and concentration was calculated. The V3-V4 hypervariable region of the bacterial 16S rRNA gene was amplified using the primers 338 F:5′- ACTCCTACGGGAGGCAGCAG-3′and 806 R:5′-GGACTACHVGGGTWTCTAAT-3′ Purified amplicons were pooled in equimolar ratios and paired-end sequenced on an Illumina MiSeq platform according to the standard protocols. The raw reads were deposited into the NCBI Sequence Read Archive (SRA) database. Raw FASTQ files were demultiplexed, quality-filtered using QIIME (version 1.91). Operational taxonomic units (OTUs) were clustered with 97% similarity cutoff using UPARSE, and chimeric sequences were identified and removed using UCHIME. Phylogenetic affiliation of each 16S rRNA gene sequence was analyzed by RDP Classifier against the Silva (SSU117/119)16S rRNA database using a confidence threshold of 70%. All the raw Illumina paired-end read data involved in this study were deposited in the NCBI SRA database under the accession number PRJNA751929. Analysis was performed at each taxonomical level (Phylum and Genus), separately. The Shannon index and Chao1 index were performed to analyze the alpha diversity. The principal coordinate analysis (PCoA) based on weighted UniFrac distance matrices was visualized for the beta diversity analysis. The dominant bacterial community difference between groups was detected using linear discriminant analysis effect size (LEfSe).

### Statistical Analyses

Statistical analysis was performed using SPSS 22.0 software. Data are expressed as the mean ± S.D. For parametric variables, differences were analyzed *via* one-way ANOVA and least significant differences (LSD) *post hoc* tests for multiple group comparisons. For nonparametric variables, statistical significance of differences among the groups was tested using the nonparametric the Kruskal–Wallis test, followed by the Mann–Whitney *U* test when *p*-value is < 0.05. A *p* < 0.05 was defined as statistically significant.

## Results

### Ginger Reduced the Susceptibility to DSS-Induced Colitis in Mice With Early-Life AZT Exposure

To estimate the long-term effect of early-life AZT exposure on DSS-induced colitis in mice and the anticolitic effect of ginger, we assessed body weight loss, DAI scores, and colon length and performed histological analysis to determine susceptibility to colitis in the mouse models.

**Figure 2:**
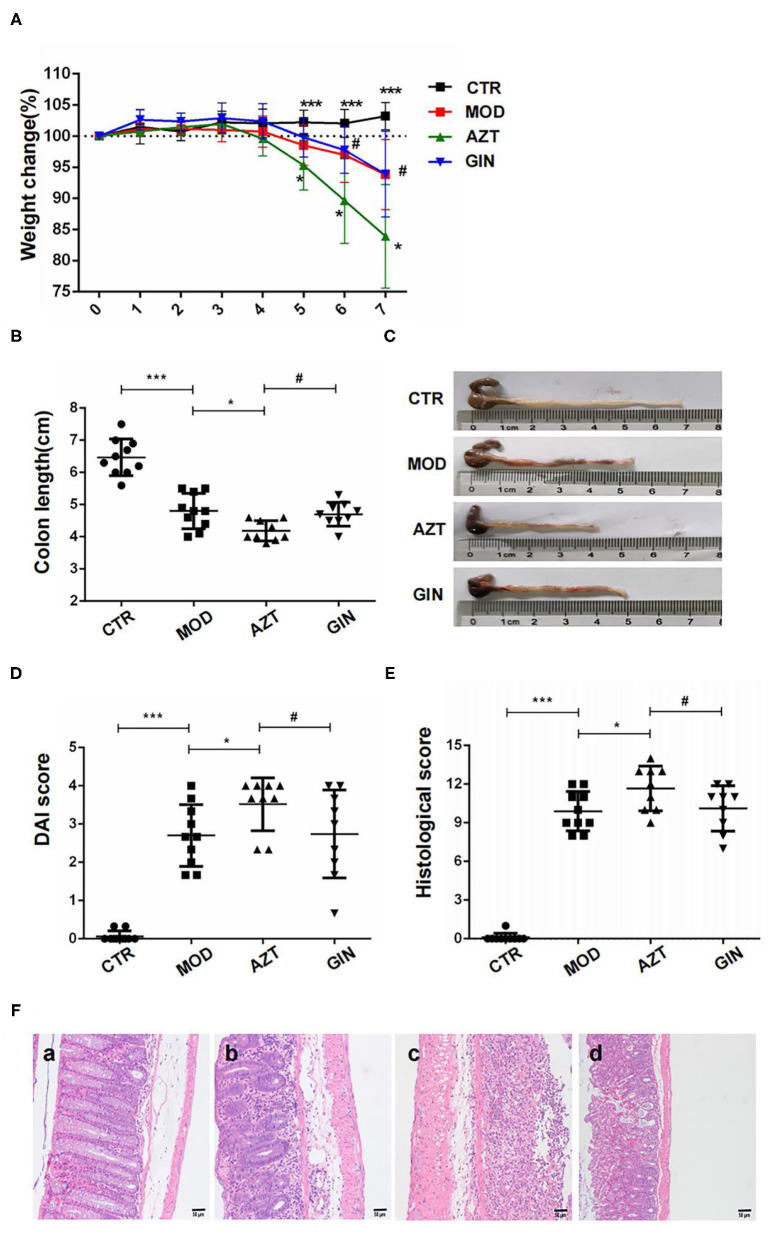
Ginger reduces the susceptibility to DSS-induced colitis in mice exposed to AZT in early life. **(A)** Body weight loss, **(B)** and **(C)** colon length, **(D)** DAI score on day 6, **(E)** histological score, and **(F)** histological examination (magnification, ×200): (a) CTR group; (b) MOD group; (c) AZT group; and (d) GIN group. Data are represented as means ± SD (*n* = 8–10). *p < 0.05 and ***p < 0.01 vs. model group; ^#^*p* < 0.05 vs. AZT group.

Compared with the control group, mice with DSS-induced colitis exhibited significant weight loss starting from day 5 until the end of DSS treatment period. Early-life AZT exposure accelerated the loss of body weight from days 5 to 7 when compared to the group treated with DSS only. However, ginger diet attenuated the progression of DSS-induced body weight loss in mice aggravated by early-life AZT exposure (Figure 2A).

Shortening of colon length in DSS-induced mice is one of the biological markers of the assessment of colonic inflammation. The colon length of the MOD group was shortened compared with that of the control group. Further, AZT treatment in early life exacerbated colonic shortening in DSS-induced colitis mice. However, ginger prevented DSS-induced colonic shortening aggravated by AZT exposure (Figures 2B,C).

The DAI scores of the MOD group were considerably increased when compared to the control group, whereas treatment with AZT in early life increased the DAI scores of DSS-induced colitis mice. In accordance with the weight loss, compared with the AZT group, mice treated with ginger after AZT exposure showed a lower DAI score (Figure 2D).

Histological characteristics of the colons were subsequently analyzed *via* H&E staining. DSS induced significant colon tissue injury, as demonstrated by loss of goblet cells, neutrophil infiltration, muscular layer thickening, goblet cell damage, and crypt distortion. AZT treatment aggravated the colon injury in DSS-induced colitis, as shown by severe transmural inflammation characterized by severe crypt damage, goblet cell loss and damage, superficial ulceration, and massive inflammatory cell infiltration and thus a significantly higher histological score than that of the model group. In turn, ginger treatment preserved the extension of crypt damage and ameliorated inflammatory reactions such as mucosal and submucosal infiltration and thus resulted in a lower histological score compared with the AZT group (Figures 2E,F).

These results indicate that early life AZT exposure promotes the development of DSS-induced colitis in adult mice. Ginger treatment decreased the susceptibility to DSS-induced colitis in mice exposed to AZT in early life.

### Ginger Improved Intestinal Barrier Function in Mice With DSS-Induced Colitis Exposed to AZT in Early Life

Intestinal epithelial tight junctions (TJs) play a key role in protecting against inflammation, and disrupted TJs are the primary cause of intestinal barrier dysfunction and inflammation. To investigate the impact of early-life AZT exposure and ginger on intestinal barrier function, we used transmission electron microscopy and immunofluorescence to analyze changes in the morphology of TJs and TJ proteins in mice with DSS-induced colitis.

**Figure 3:**
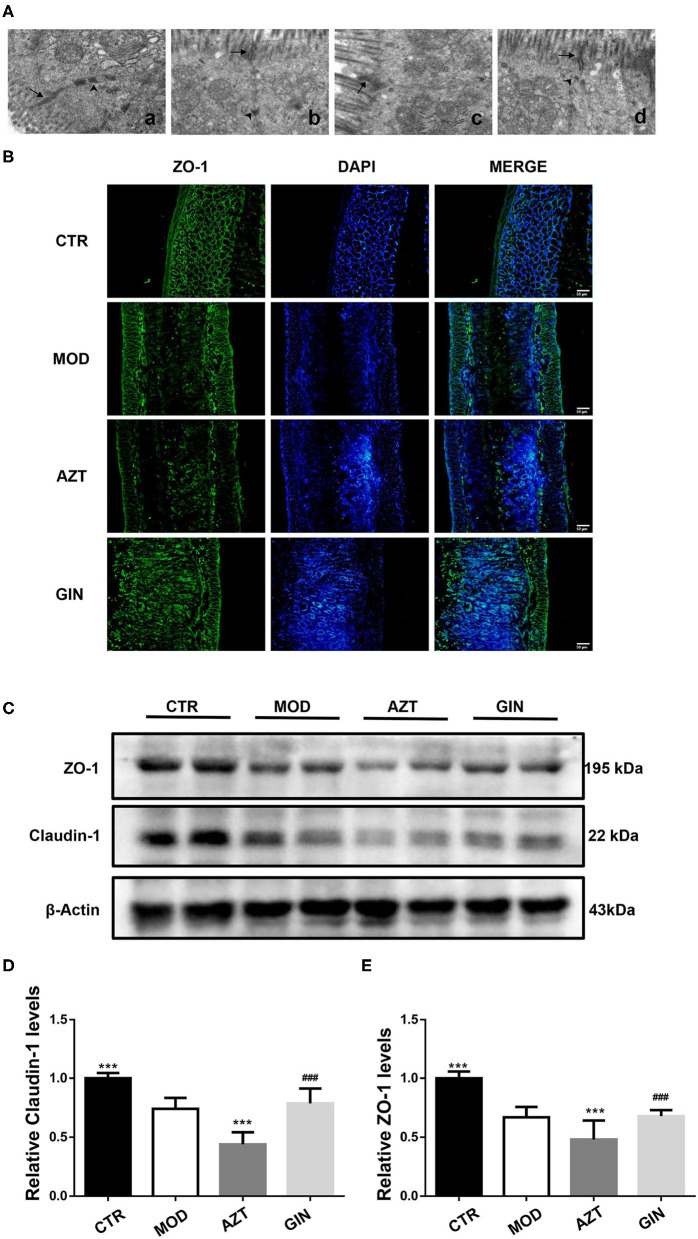
Ginger improves the intestinal barrier function of mice with DSS-induced colitis exposed to AZT in early life. **(A)** TJ morphology (magnification, ×8000): (a) CTR group; (b) MOD group; (c) AZT group; and (d) GIN group. Arrows, TJ; arrow heads, desmosome. **(B)** Representative images of immunofluorescence of ZO-1 in colon sections. **(C)** Western blot analysis of claudin-1 and ZO-1 expression in colon tissue. **(D)** and **(E)** Relative levels of claudin-1 and ZO-1 (*n* = 4). ***p < 0.01 vs. MOD group; ^###^*p* < 0.01 vs. AZT group.

In control mice, the TJs appeared as typical membrane fusions with intact TJ structure and desmosomes. In contrast, the TJ ultrastructure was altered after DSS treatment. TJs were discontinuous with few membrane fusions apparent in colon tissues, which indicated disruption in TJ morphology. Compared with the model group, the AZT-treated mice showed altered TJ ultrastructure. The TJ showed few electron-dense materials and loss of desmosome. However, treatment with ginger restored the TJ ultrastructure disrupted by early-life AZT exposure (Figure 3A).

The distribution of tight junctional proteins, ZO-1 and Claudin-1, which are the markers of TJ structure, was analyzed *via* immunostaining and the Western blot. Compared with control mice, the expression of colonic ZO-1 and claudin-1 was significantly decreased in MOD group mice and further decreased in AZT group (Figures 3B–E). Ginger treatment upregulated the expression of TJ proteins in mice exposed to AZT in early life.

### Ginger Inhibited Inflammatory Response in DSS-Induced Colitis of Mice With Early-Life AZT Exposure

**Figure 4:**
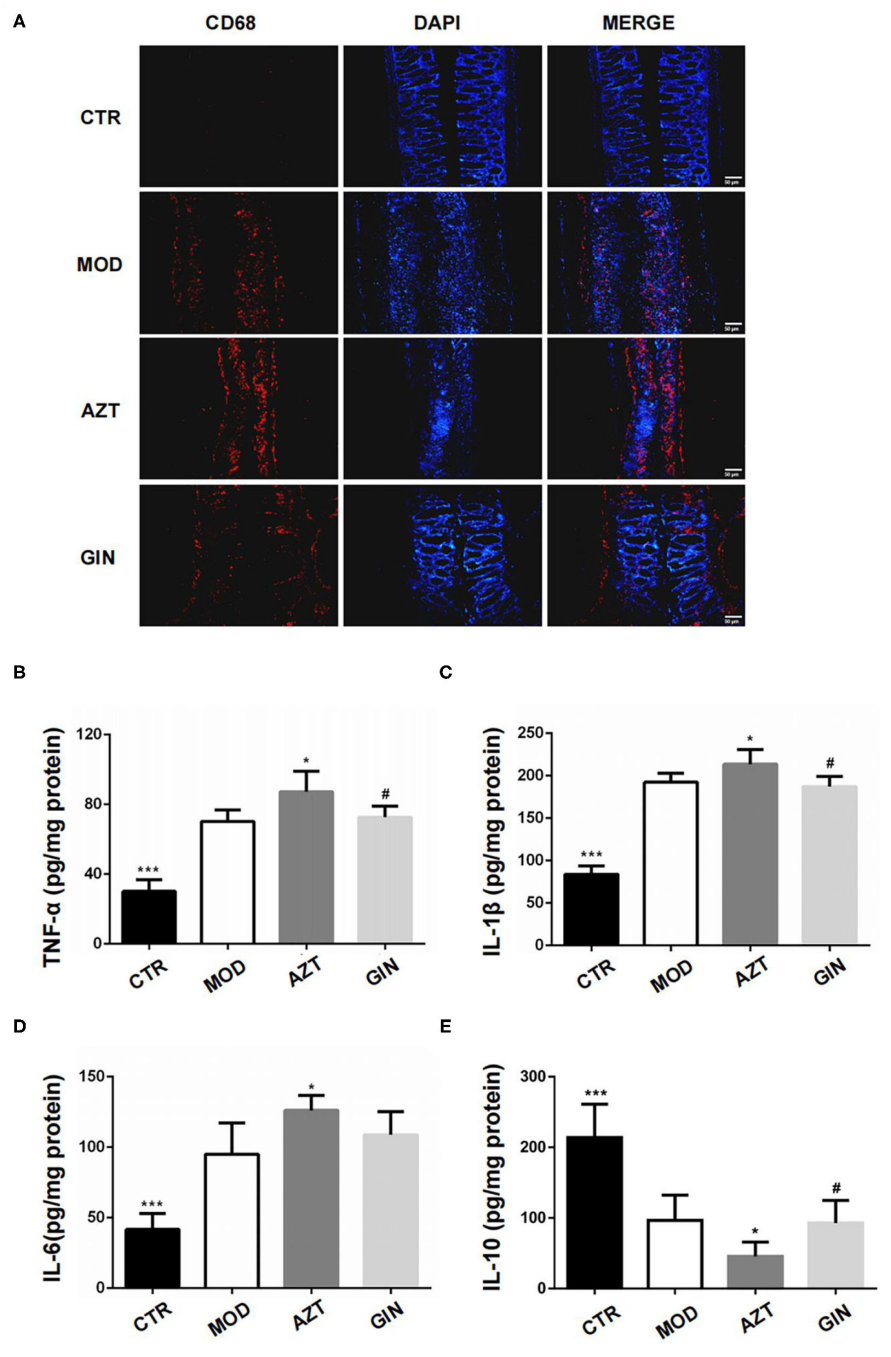
Ginger inhibits inflammatory response in DSS-induced colitis mice exposed to AZT in early life. **(A)** Representative images of immunofluorescence of CD68-positive macrophages in colon sections. **(B–E)** Colonic cytokine levels of TNF-α, IL-1β, IL-6, and IL-10. Data represent means ± SD (*n* = 5). *p < 0.05 and ***p < 0.01 vs. MOD group; ^#^*p* < 0.05 vs. AZT group.

Inflammatory bowel disease is characterized by infiltration of immune cells and elevated levels of proinflammatory factors. We first investigated the influence of early AZT exposure on colonic macrophages, since they were easily activated after disruption of intestinal epithelial barrier function. CD68 was used as a marker of macrophages in the colon of mice in this study. Immunofluorescence revealed increased infiltration of macrophages in colonic lamina propria of mice treated with DSS, and macrophages were further increased in AZT group. Treatment with ginger inhibited the infiltration of macrophages in DSS-induced colitis associated with early AZT exposure (Figure 4A).

Colonic inflammatory cytokines, including TNF-α, IL-1β, IL-6, and IL-10, were measured using ELISA kits. The colitis model group expressed significantly higher levels of colonic TNF-α, IL-1β, and IL-6, and lower levels of IL-10 compared with the control group. The AZT group showed further increase of TNF-α, IL-1β, and IL-6 and a further decrease in IL-10 levels. Compared with AZT group, the group treated with ginger extract showed suppression of TNF-α and IL-1β levels and the expression of IL-10 (Figures 4B–E).

### Ginger Modulated Intestinal Microbiota Dysbiosis Induced by Early-Life AZT Exposure

Gut microbiota has emerged as an important factor in the development and function of the immune system. As antibiotics directly influence gut microbiota, we performed 16S rRNA gene sequencing to determine the impact of early-life AZT exposure and ginger on gut microbiota in later life. Fecal samples derived from the model group, AZT group, and GIN group were collected before DSS treatment. The control and model groups were exposed to similar treatment, and the model group alone was selected for the analysis.

**Figure 5:**
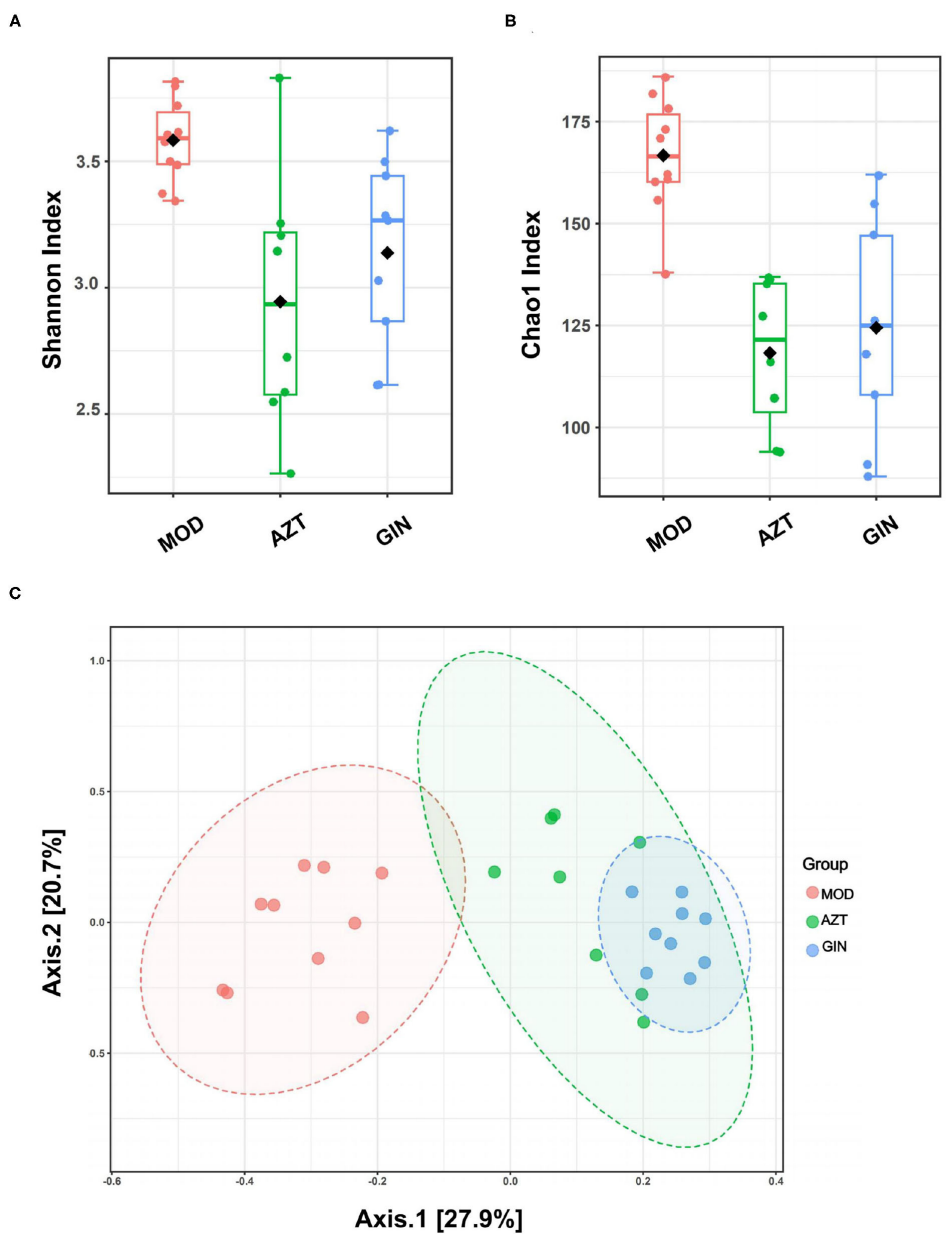
Ginger modulates the diversity and structure of gut microbiota induced by early AZT exposure. Comparison of alpha diversity based on **(A)** Shannon index and **(B)** Chao1 index. **(C)** Multisample PCoA (*n* = 8–10).

Results showed that early-life AZT exposure significantly decreased the alpha diversity of bacteria, and treatment with ginger reversed it, based on Shannon index and Chao1 index (Figures 5A,B). Beta diversity represented by PCoA is shown in Figure 5C. The results revealed that the gut microbiota in the AZT group deviated from the baseline structure. Ginger modulated the effect on dysbiosis although it failed to completely restore the microbiota to normal status.

**Figure 6:**
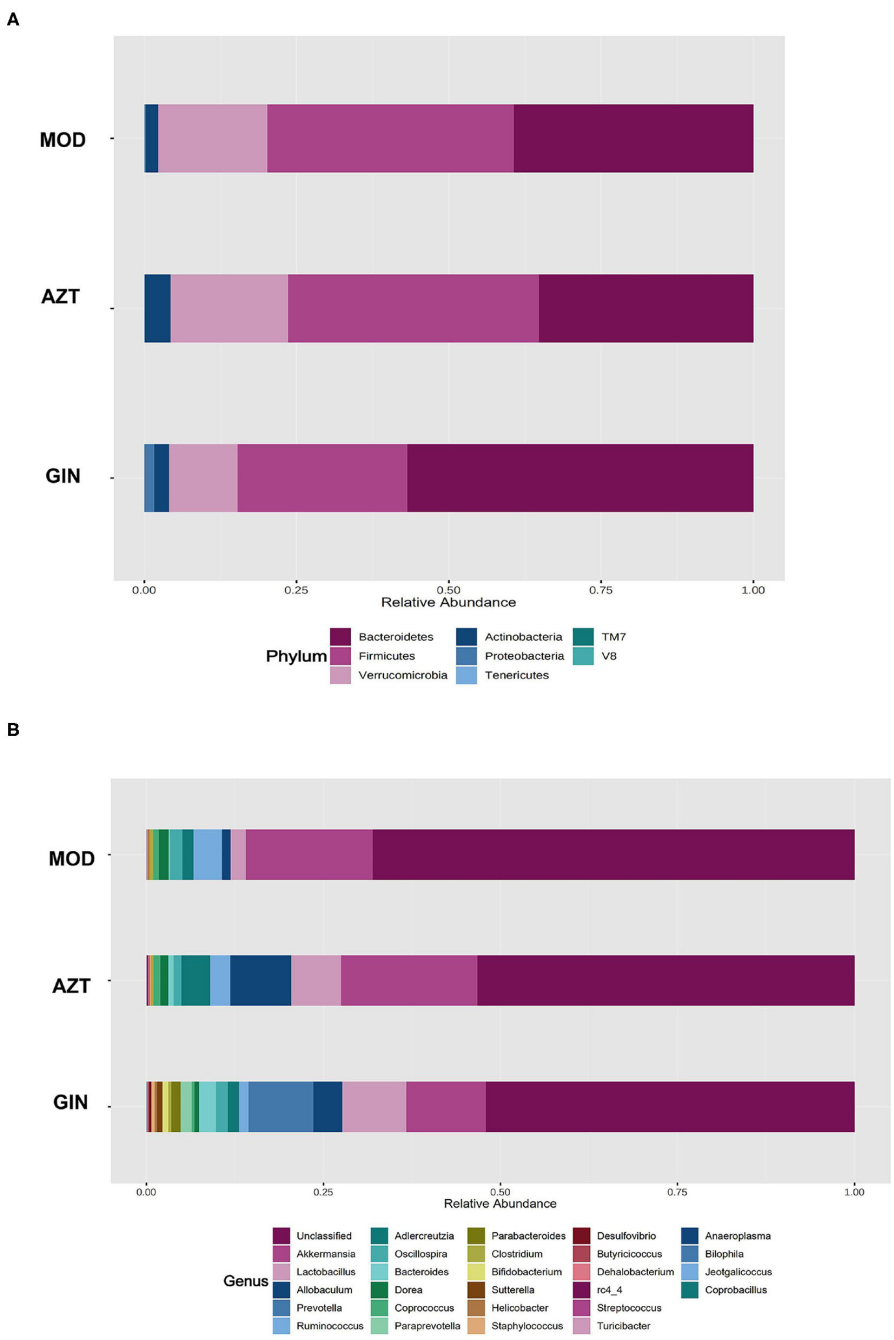
Ginger modulates the composition of gut microbiota induced by early AZT exposure. **(A)** Microbial community bar plot by phylum. **(B)** Microbial community bar plot by genus.

**Figure 7:**
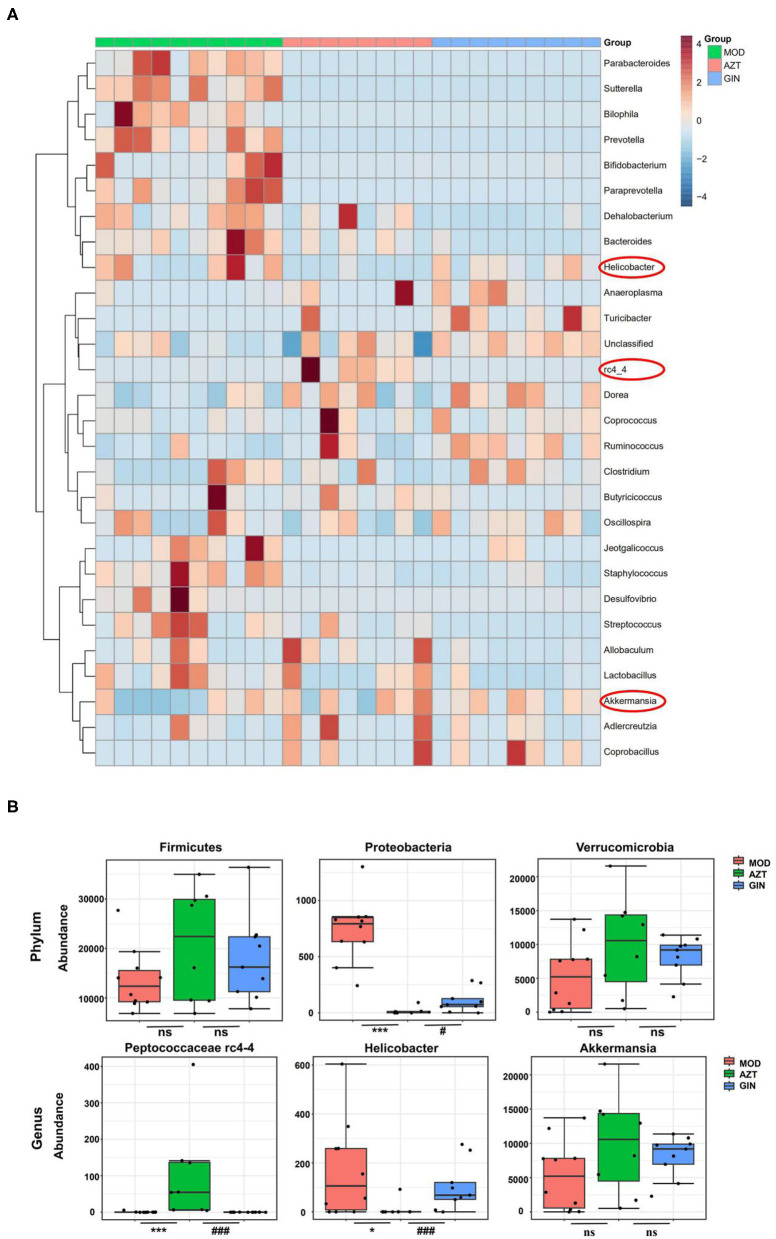
Ginger modulates the composition of gut microbiota induced by early AZT exposure. **(A)** Heatmap of the relative abundance of key 27 OTUs altered by AZT and ginger at genus level. **(B)** The relative abundance of microbial species at phylum and genus level. *p < 0.05 and ***p < 0.01 vs. MOD group; ^#^*p* < 0.05 and ^###^*p* < 0.01 vs. AZT group.

Further, early-life AZT exposure altered the composition of the microbiota, and ginger modulated the dysbiosis. Changes were observed at the levels of both phylum and genus. At the phylum level, a total of seven phyla were identified, including Bacteroidetes, Firmicutes, Verrucomicrobia, Actinobacteria, Proteobacteria, Tenericutes, and TM7 (Figure 6A). At the genus level, 27 genera (Supplementary Table S4) including *Akkermansia, Helicobacter, Peptococcus* rc4-4, *Lactobacillus*, and other genera were identified (Figure 6B). The relative abundance of Proteobacteria decreased and the relative abundance of Firmicutes and Verrucomicrobia increased in the AZT group compared with the model group. The changes in Firmicutes, Verrucomicrobia, and Proteobacteria induced by early AZT exposure were reversed by ginger treatment, although no statistically significant differences were detected in Firmicutes and Verrucomicrobia (Figure 7B) The changes in the main microbiota at the genus level were captured using a heatmap of 27 key species with varying degrees of abundance among the groups. Early AZT exposure not only eliminated harmful bacteria, including *Staphylococcus, Desulfovibrio*, and *Streptococcus*, but also decreased the abundance of beneficial bacteria, including *Lactobacillus, Bifidobacterium, and Parabacteroides* (Figure 7A). However, the levels of most of these reduced genera could not be restored to the normal level after ginger treatment. Notably, the relative abundance of *Helicobacter* decreased and the relative abundance of Peptococcaceae rc4-4 increased significantly in the AZT group compared with the model group. These changes induced by early AZT exposure were reversed by ginger treatment (Figure 7B). In addition, early antibiotic exposure also increased the relative abundance of *Akkermansia*, and ginger treatment had a modulatory effect without statistical significance.

A schematic diagram of evolutionary clustering analysis was generated taxonomically based on the LDA score to identify key microbiota responsible for the differences among groups. As shown in Supplementary Figures S2A,B, *Bacteroidia, Helicobacter*, and *Desulfovibrio* were the major microbiota in MOD group. Species belonging to Peptococcaceae and Turicibacter were identified as major microbiota in the AZT group. In GIN group, the predominant intestinal flora, including *Akkermansia muciniphila* and *Anaerostipes*, especially *A. muciniphila*, may play an important role in DSS-induced colitis of mice exposed to AZT in early life.

## Discussion

Mice exposed to AZT early in life were highly susceptible to DSS-induced colitis and were successfully treated with ginger in this study. A mouse model mimicking childhood antibiotic exposure in humans was used for the first time. DSS treatment induced clinical symptoms and pathological changes corresponding to those of human UC, including loss of body weight, diarrhea, bloody feces, and shortening of the colon. The measurement of body weight loss is a standard method used to evaluate disease progression in DSS-induced colitis. Weight loss and rectal bleeding are connected with colon shortening. Early AZT exposure significantly exacerbated DSS-induced colitis by decreasing weight loss, DAI scores, and colonic shortening. Treatment with ginger ameliorated these symptoms and histopathology effectively. In contrast, administration of ginger after early AZT exposure reduced all of the aforementioned inflammatory changes, suggesting that early AZT exposure exacerbated the inflammatory condition induced by DSS, which was ameliorated by treatment with ginger.

Intestinal epithelium constitutes a physical and functional barrier separating lamina propria from luminal pathogens and antigen exposure. It serves as a first line of defense for the mucosal immune system. Compromised intestinal barrier function is associated with the development of IBD. The TJ proteins are crucial for the maintenance of epithelial barrier integrity. ZO-1 and occludin represent TJ proteins playing a pivotal role in regulating paracellular permeability. In this study, compared with the MOD group, the AZT group decreased ZO-1 and occludin membrane localization in the colon. A previous study showed that ginger enhanced the expression of ZO-1 and occludin in the TJ of colonic mucosa in mice with colitis (20). Similarly, the expression of ZO-1 and occludin was recovered after ginger treatment. These results indicate the detrimental effects of early antibiotic exposure and the protective role of ginger in DSS-induced disruption of the intestinal epithelial barrier.

Disruption of intestinal epithelial barrier function may lead to translocation of intestinal bacteria and entry of gut-derived pathogens into portal circulation through the highly permeable intestinal barrier. The consequences include activated nonlymphoid cells, such as macrophages and increased proinflammatory cytokines, including TNF-α, IL-6, and IL-1β. Macrophages are the key regulators of immune response and critical for maintaining immunological homeostasis in the intestine. A previous study demonstrated that antibiotic-induced bacterial disturbances led to persistent changes in adaptive immunity in the intestine by interfering with microbiota-dependent regulation of intestinal macrophage function (21). Here, we also found that early antibiotic exposure increased infiltration of intestinal macrophages and proinflammatory cytokines in DSS-induced colitis of mice in later years. Consistent with previous studies (15, 20), ginger treatment after antibiotic exposure reduced the levels of TNF-α, IL-6, and IL-1β in the colon and decreased macrophage infiltration to inhibit inflammation.

Gut microbiota are the key factors in IBD pathogenesis, as colitis cannot be induced by DSS in germ-free animals and many IBD susceptibility genes are associated with host-microbe immune interactions (22). We then analyzed the impact of early antibiotic exposure on gut microbiota as a possible mechanism in aggravating colitis. The decline in microbial diversity was shown in AZT group, and the diversity was improved after ginger treatment. The PCoA showed the differential composition of bacterial communities in DSS and AZT groups. Ginger modulated the effect on dysbiosis, but still failed to restore microbiota to normal condition. The gut microbiota communities in all samples were evaluated based on phylum and genus. In terms of bacterial composition at the phylum level, early AZT exposure increased the relative abundance of Firmicutes and Verrucomicrobia and decreased the relative abundance of Proteobacteria. Treatment with ginger reversed the changes in Firmicutes, Proteobacteria, and Verrucomicrobia. At the genus level, early AZT exposure not only killed harmful bacteria, including *Staphylococcus, Desulfovibrio, and Streptococcus*, but also decreased the abundance of beneficial bacteria, including *Lactobacillus and Bifidobacterium*. The modulatory effect on bacteria mentioned above was weak, but ginger treatment reversed the increased abundance of Peptococcaceae rc4-4 and the decreased abundance of *Helicobacter* significantly induced by early AZT exposure.

*Peptococcus*, a type of intestinal sulfate-reducing bacteria (SRB), (23) was more prevalent in mice exposed to AZT early, but decreased significantly in the ginger-treated group. Although little is known about *Peptococcaceae*, SRB have been found to generate hydrogen sulfide (H2S), which is toxic to colonic epithelial cells by inhibiting butyrate metabolism in colonocytes and contributes to inflammation in experimental colitis (24). Previous studies also showed that SRB reduced the mucus barrier function and facilitated bacterial presence close to the colonic epithelium, leading to inflammation (25). These findings suggest that *Peptococcaceae* may be important for the development of colitis.

*Helicobacter pylori* has been characterized as the primary pathogenic factor in chronic gastritis and peptic ulcer. However, the relative abundance of *Helicobacter* was increased significantly in the AZT group but decreased in the ginger-treated group. Interestingly, numerous studies have reported a lower level of *Helicobacter pylori* infection rate in IBD patients than in controls (26, 27). Animal experiments also confirmed the inverse association between *Helicobacter pylori* and IBD. *H. pylori* DNA significantly ameliorated colitis and histopathological changes in a DSS-induced mouse colitis model (28). Epidemiological and basic experimental studies suggested that Helicobacter infection protects against IBD by inducing systematic immune tolerance and suppressing inflammatory responses (29). Further evidence indicates that children and youth with developing immune systems may benefit more than older individuals from immune tolerance induced by *Helicobacter* (30, 31). These studies underscore the need for caution in eradicating *H. pylori*, especially for children.

Interestingly, we also found that the relative abundance of *Akkermansia* in five of eight samples was increased after early AZT exposure, though there was no statistical difference. Mucin-degrading *A. muciniphila* is a commensal bacterium dwelling in the mammalian gastrointestinal tract adhering to the mucus layer and plays an essential role in maintaining gut barrier function. The presence of *Akkermansia* has also been reported in other antibiotic treatment models (32). Recent studies about *Akkermansia* indicate divergent host outcomes including improved metabolic phenotypes and enhanced lifespan (33, 34). However, the enrichment of *Akkermansia* has also been associated with increased susceptibility to colitis (35). *Akkermansia* is sufficient for promoting intestinal inflammation in both specific-pathogen-free and germ-free IL10^−/−^ mice, representing models of spontaneous colitis (36). These findings suggested that *Akkermansia* may exhibit different or even contrasting physiological role in acute colitis models. In our mouse model, early antibiotic exposure induced a bloom of *Akkermansia*, which may have contributed to the aggravation of colitis. In turn, ginger treatment decreases the abundance of *Akkermansia*, indicating its modulation effect on microbiota of mice exposed to antibiotics early in life.

Therefore, early antibiotic exposure may lead to sustained dysbiosis that increases the susceptibility to colitis later, and ginger treatment modulates these changes effectively. This study extends our understanding of the impact of early-life antibiotic exposure with respect to microbial dysbiosis and colitis, and interventions *via* ginger targeting gut microbiota to ameliorate these changes.

However, this study still has certain limitations. First, the main active component in ginger extract that contributes to antiinflammatory effects and microbial regulation requires further investigation. Second, the specific bacterial species mediating susceptibility of murine colitis in mice exposed to AZT early in life remains to be verified in the future.

## Data Availability Statement

The data presented in the study are deposited in the NCBI SRA repository, accession number: PRJNA751929.

## Ethics Statement

The animal study was reviewed and approved by Animal Ethics Committee of Guangzhou Institute of Sport Science (GZTKSGNX-2016-4). Written informed consent was obtained from the owners for the participation of their animals in this study.

## Author Contributions

XZho, XL, and QH conceived the research idea. XZho, XL, QH, and HL performed the experiments. YY, LC, and MW provided technical and material support. XZho and QH analyzed the data. XZho prepared the manuscript. LZ, XF, and HK revised the manuscript. LZ and XZha reviewed the manuscript. All authors contributed to the article and approved the final manuscript.

## Funding

This work was supported by the National Key R&D Program of China (2020YFC2003100 and 2020YFC2003101), the Key Project of National Natural Science Foundation of China (81830117), the National Natural Science Foundation of China (81774212 and 81760821), the Natural Science Foundation of Guangdong Province, China (2019A1515010400), the Science and Technical Plan of Guangzhou, Guangdong, China (201903010069), and the Innovation Team and Talents Cultivation Program of the National Administration of Traditional Chinese Medicine (Number: ZYYCXTD-C-202001).

## Conflict of Interest

The authors declare that the research was conducted in the absence of any commercial or financial relationships that could be construed as a potential conflict of interest.

## Publisher's Note

All claims expressed in this article are solely those of the authors and do not necessarily represent those of their affiliated organizations, or those of the publisher, the editors and the reviewers. Any product that may be evaluated in this article, or claim that may be made by its manufacturer, is not guaranteed or endorsed by the publisher.
