# Supplementary material for: Ginger Extract Decreases Susceptibility to Dextran Sulfate Sodium-Induced Colitis in Mice Following Early Antibiotic Exposure
Source: Front Med (Lausanne). 2022 Jan 5;8:755969. doi: 10.3389/fmed.2021.755969 (PMC8766511; doi:10.3389/fmed.2021.755969)
Supplement: Supplementary file 1 [file Data_Sheet_1.docx]

Supplementary Material

**
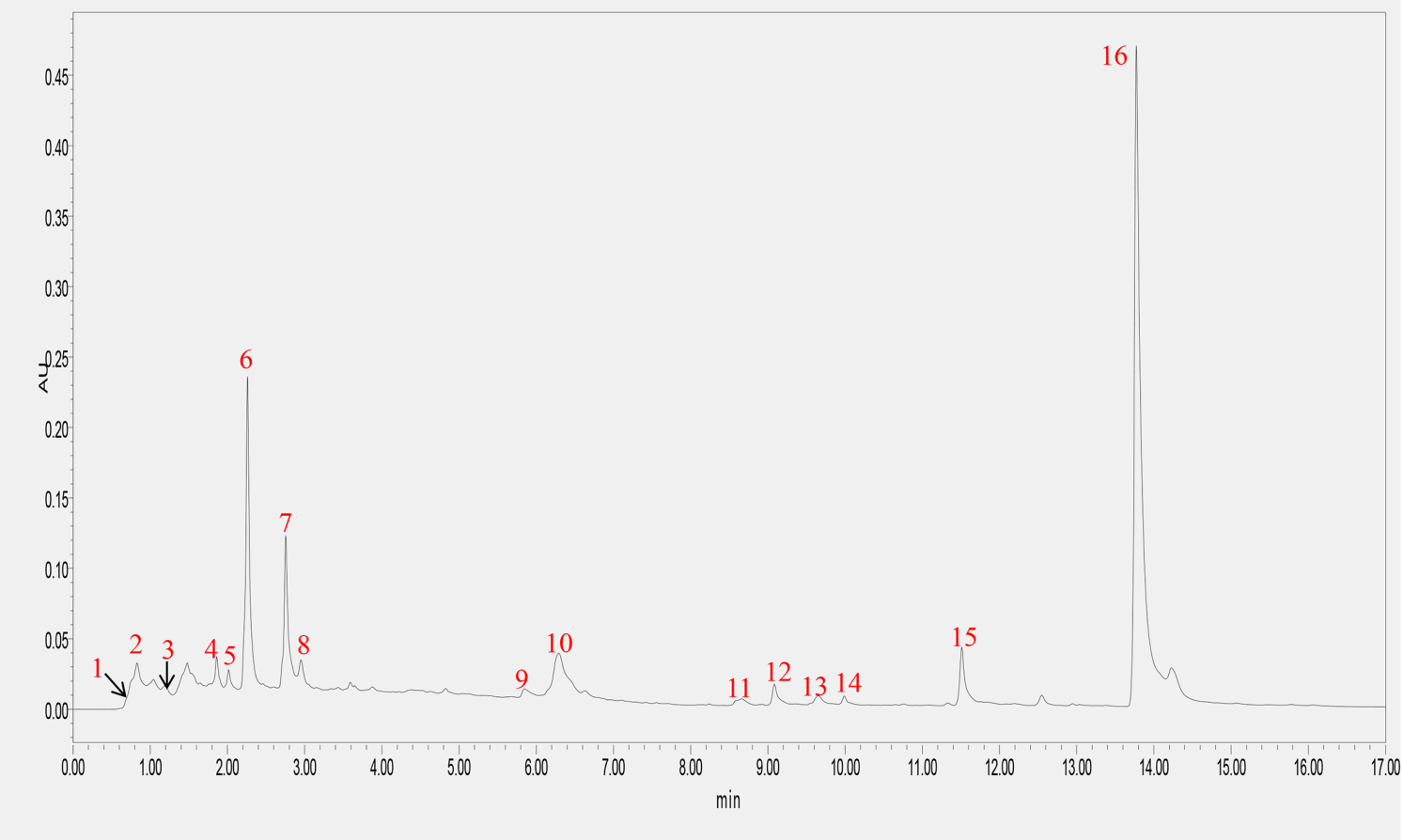
**

**Fig. S1.** UPLC-QTOF-MS analysis of ginger extract. Representative UPLC chromatograms of ginger extract are shown. The identified compounds are represented by numbers(**Table S1**).

**
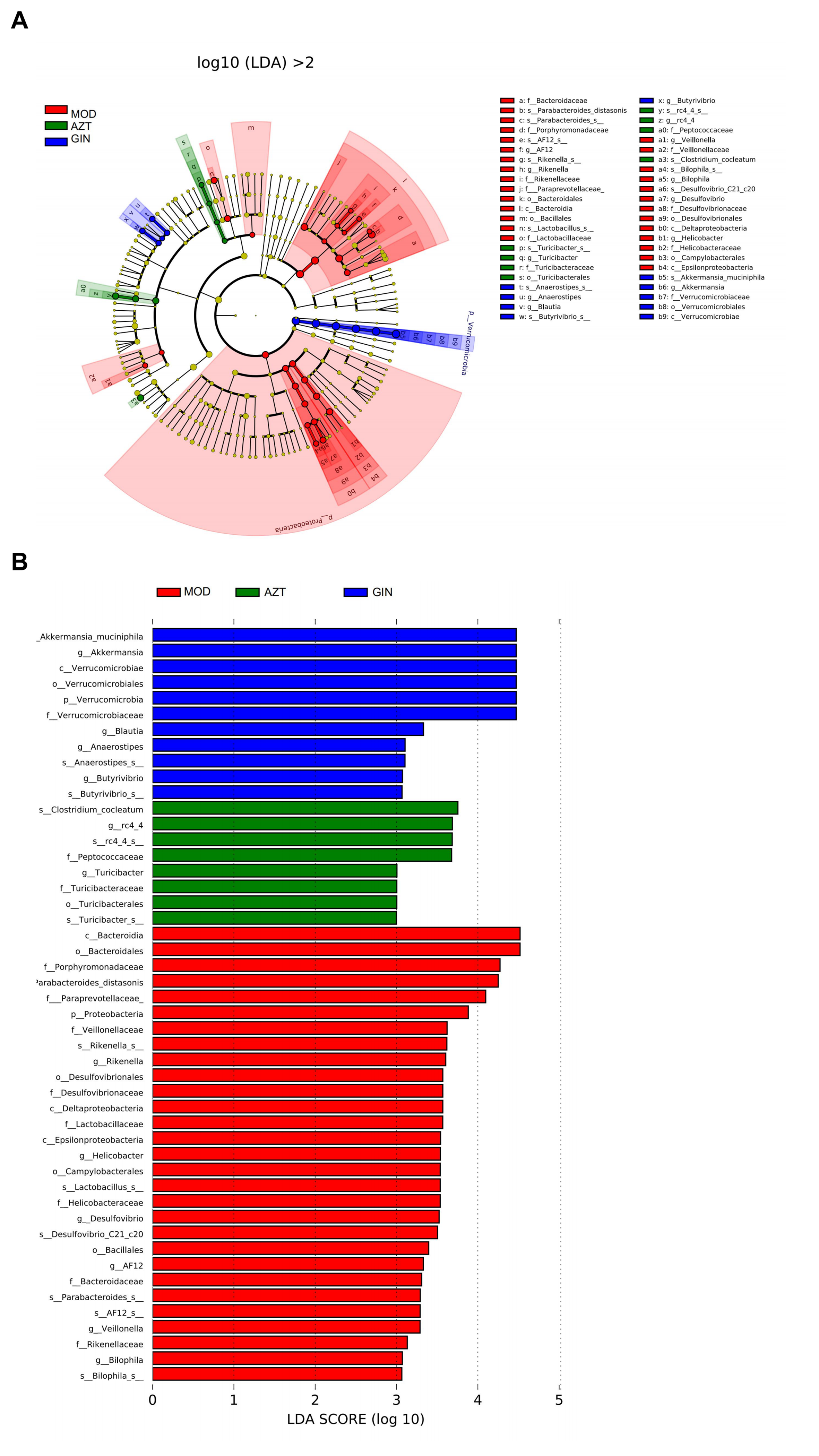
**

**Figure S2.** Linear discriminant analysis Effect Size **(**LEfSe) approach is used to identify the key phylotypes underlying the differences between groups. (A) Cladogram. (B) Distribution histogram based on LDA.

**Table S1. Compounds identified in ginger extract by HPLC-Q-TOF-MS/MS**

| Peak | Rt(min) | m/z | Adduct | Compounds |
| --- | --- | --- | --- | --- |
| 1 | 0.68 | 341.1145 | M-H | Turanose |
| 2 | 0.82 | 133.0163 | M+H | 2-Deoxy-D-Ribose |
| 3 | 1.08 | 130.0483 | M+H | Pyroglutamic acid |
| 4 | 1.81 | 166.0848 | M+H | Phenylalanine |
| 5 | 1.93 | 125.0264 | M-H | Thymine |
| 6 | 2.51 | 291.015 | M+H | 6-Dehydrogingerdione |
| 7 | 2.9 | 395.1993 | M+COOH | 10-Gingerol |
| 8 | 3.07 | 363.172 | M-H | Methyl-10-Gingerol |
| 9 | 5.92 | 389.1672 | M-H | Acetoxy-10-Gingerol |
| 10 | 6.55 | 503.2595 | M+COOH | 6-Gingerdiol 5-O-beta-D-glucopyranoside |
| 11 | 8.79 | 527.1312 | M+COOH | O-tert-Butyl-dimethylsilyl Curcumin |
| 12 | 9.18 | 351.0453 | M+COOH | 8-Paradol |
| 13 | 9.26 | 381.1575 | M+H | Diacetoxy-6-gingerdiol |
| 14 | 9.72 | 277.1788 | M+H | 6-Shogaol |
| 15 | 11.6 | 407.172 | M+H | 14-gingerol |
| 16 | 13.63 | 279.1575 | M+H | 6-Paradol |

**Table S2. Evaluation of disease activity index (DAI)**

| **DAI score** | **Weight loss (%)** | **Stool consistency** | **Stool bleeding** |
| --- | --- | --- | --- |
| 0 | None | Normal | Negative |
| 1 | 1-5 |  | + |
| 2 | 5-10 | Loose | ++ |
| 3 | 10-15 |  | +++ |
| 4 | >15 | Diarrhea | ++++ |

**Table S3. Histologic scores of colitis**

| **Histological parameters** | **Description** | **Score** |
| --- | --- | --- |
| Degree of inflammation | none | 0 |
|  | slight | 1 |
|  | moderate | 2 |
|  | severe | 3 |
| extent | none | 0 |
|  | mucosa | 1 |
|  | mucosa and submucosa | 2 |
|  | transmural | 3 |
| Crypt damage | none | 0 |
|  | basal 1/3 damage | 1 |
|  | basal 2/3 damage | 2 |
|  | only surface epithelium intact | 3 |
|  | entire crypt and epithelium lost | 4 |
| Percent involvement | 1–25% | 1 |
|  | 26–50% | 2 |
|  | 51–75% | 3 |
|  | 76–100% | 4 |

**Table S4. Twenty-seven genera identified in mouse faeces**

| **Genus** | **Genus** | **Genus** |  |
| --- | --- | --- | --- |
| *Akkermansia* | *Coprococcus* | *Butyricicoccus* |  |
| *Lactobacillus* | *Parabacteroides* | *Dehalobacterium* |  |
| *Allobaculum* | *Paraprevotella* | *Peptococcaceae rc4-4* |  |
| *Prevotella* | *Clostridium* | *Streptococcus* |  |
| *Ruminococcus* | *Bifidobacterium* | *Turicibacter* |  |
| *Adlercreutzia* | *Sutterella* | *Anaerostipes* |  |
| *Oscillospira* | *Helicobacter* | *Bilophila* |  |
| *Bacteroides* | *Staphylococcus* | *Jeotgalicoccus* |  |
| *Dorea* | *Desulfovibrio* | *Coprobacillus* |  |
