# Supplementary material for: Ginger Extract Decreases Susceptibility to Dextran Sulfate Sodium-Induced Colitis in Mice Following Early Antibiotic Exposure
Source: Front Med (Lausanne). 2022 Jan 5;8:755969. doi: 10.3389/fmed.2021.755969 (PMC8766511; doi:10.3389/fmed.2021.755969)
Supplement: Supplementary file 2 [file Data_Sheet_2.docx]

All the source data were upload to *https://www.jianguoyun.com/.*

The download links are as follows:

**Figure2**:*https://www.jianguoyun.com/p/DWXMrdwQ4s7eCRjii4YE*

**Figure3**:*https://www.jianguoyun.com/p/DctzO78Q4s7eCRjei4YE*

**Figure4**:*https://www.jianguoyun.com/p/DWlQhnsQ4s7eCRjfi4YE*

**Figure5-7 and S2**: *https://www.jianguoyun.com/p/DdJvI8EQ4s7eCRiKiZUE*

**Figure S1**:*https://www.jianguoyun.com/p/De83N5sQ4s7eCRiLiZUE*
